# Brief data overview of differently heat treated binder jet printed samples made from argon atomized alloy 625 powder

**DOI:** 10.1016/j.dib.2016.09.042

**Published:** 2016-09-30

**Authors:** Amir Mostafaei, Yashar Behnamian, Yuval L. Krimer, Erica L. Stevens, Jing Li Luo, Markus Chmielus

**Affiliations:** a Department of Mechanical Engineering and Materials Science, University of Pittsburgh, Pittsburgh, PA 15261, USA; b Department of Chemical and Materials Engineering, University of Alberta, Edmonton, Alberta T6G 1H9, Canada

**Keywords:** Additive Manufacturing, Powder Bed Binder Jet Printing, Powder Analysis, Mechanical Properties, Microstructure, Sintering, Aging, Solutionizing

## Abstract

Powder bed binder jet printing (BJP) is an additive manufacturing method in which powder is deposited layer-by-layer and selectively joined in each layer with binder. The data presented here relates to the characterization of the as-received feedstock powder, BJP processing parameters, sample preparation and sintering profile (“Effect of solutionizing and aging on the microstructure and mechanical properties of powder bed binder jet printed nickel-based superalloy 625” (A. Mostafaei, Y. Behnamian, Y.L. Krimer, E.L. Stevens, J.L. Luo, M. Chmielus, 2016) [1], “Powder bed binder jet printed alloy 625: densification, microstructure and mechanical properties” (A. Mostafaei, E. Stevens, E. Hughes, S. Biery, C. Hilla, M. Chmielus, 2016) [2]). The data presented here relates to the characterization of the as-received feedstock powder, BJP processing parameters, sample preparation and sintering profile. Effect of post heat treatments including solutionizing and aging on the microstructure and mechanical properties of powder bed binder jet printed nickel-based superalloy 625 were compared to that of sintered samples.

**Specifications Table**

**Table:** 

Subject area	*Materials Science and Engineering*
More specific subject area	*Additive Manufacturing of nickel superalloy*
Type of data	*Figures*
How data was acquired	*Images taken from M-Flex ExOne machine were captured at the Additive Manufacturing Center at the University of Pittsburgh. Characterizations of powder and BJP samples were conducted using a laser particle analyzer, a differential scanning calorimeter (DSC), a scanning electron microscope (SEM), X-ray diffraction patterns (XRD), and microhardness and tensile tests.*
Data format	*Analyzed*
Experimental factors	*An M-Flex ExOne powder bed binder jet printer was used to produce alloy 625 parts with the following printing parameters: layer height of 100* *μm, recoat speed of 130* *mm/s, oscillator speed of 2050 rpm, roller speed of 250 rpm, roller traverse speed of 15* *mm/s, and drying speed of 17* *mm/s.*
Experimental features	*After completing the printing process, the resulting semi-finished products (“green parts”) were cured at 175 °C in a JPW Design & Manufacturing furnace and then sintered in a Lindberg tube furnace in an alumina powder bed under vacuum with the following heating profile: heating at 5 °C/min from RT to 600 °C, 3.2 °C/min to 1000 °C, 2.8 °C/min to the holding temperature (1200 °C, 1240 °C and 1280 °C), holding for 4 h and then cooling at 1 °C/min to 1200 °C, 3.1 °C/min to 500 °C and finally to RT with a temperature stability of 1 °C. Moreover, sintered sample with highest density were solution treated at 1150 °C for 2 h, and then additionally aged at 745 °C for 20 or 60 h*[1], [2].
Data source location	*University of Pittsburgh, Pittsburgh, Pennsylvania, United States*
Data accessibility	*Data is with the article*

**Value of the data**

• The printing parameters presented here may help to obtain the highest green part density of the BJP part of other nickel-based superalloys.
• Other researchers can use the presented data as a guideline in printing, sintering and post heat treatment of the additive manufactured part.
• Data allow one to ascertain process-property relationships for binder jet printed parts, providing a means to relate observed microstructural features with experimentally measured mechanical properties of the additively-manufactured samples.
• The same procedure can be followed for other types of nickel-based superalloys.

## 1 Data

**Fig. 1:**
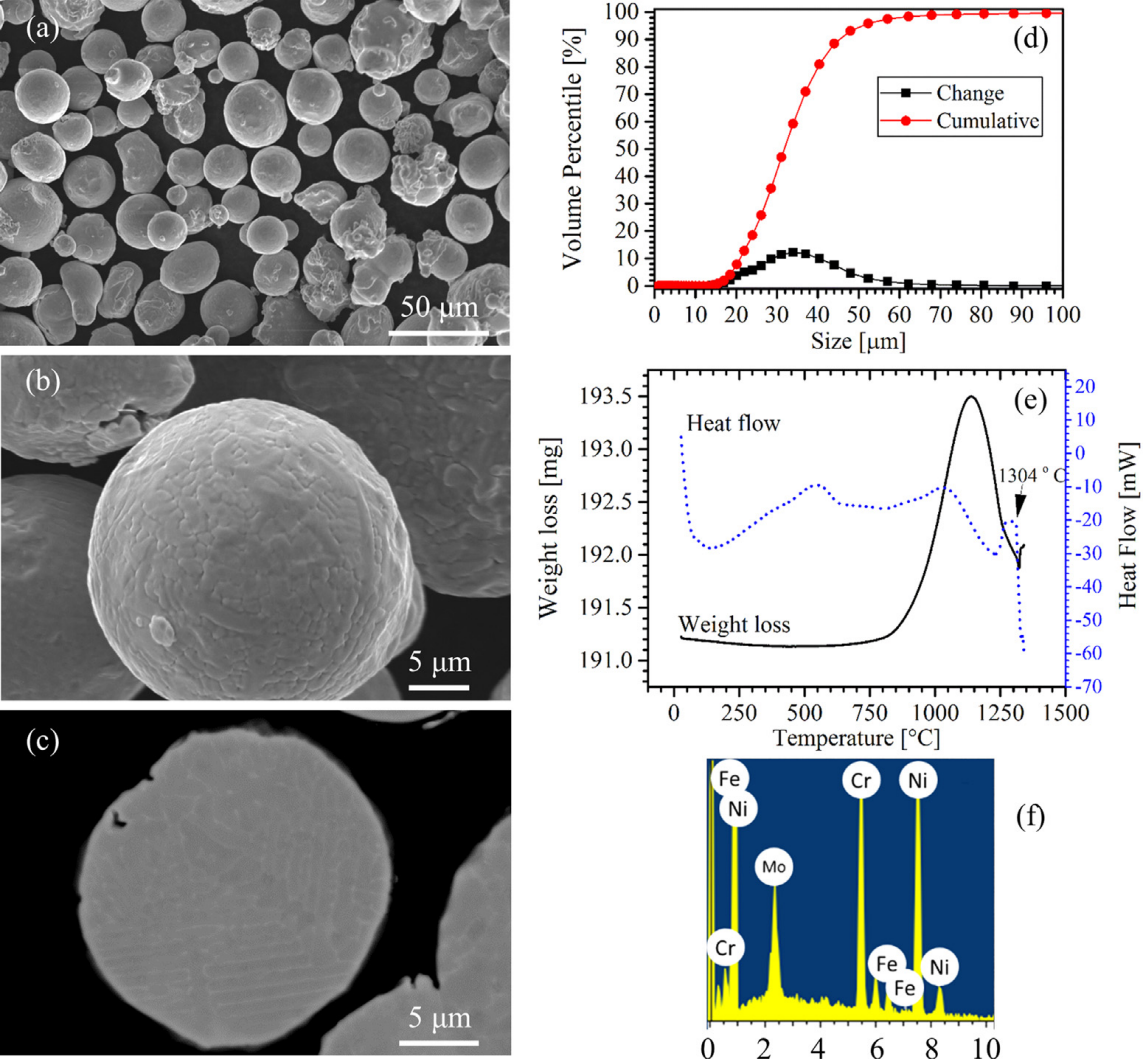
(a–c) Powder morphology micrographs, (d) particle size distribution, (e) DSC-TGA curves and (f) EDS elemental analysis.

**Fig. 2:**
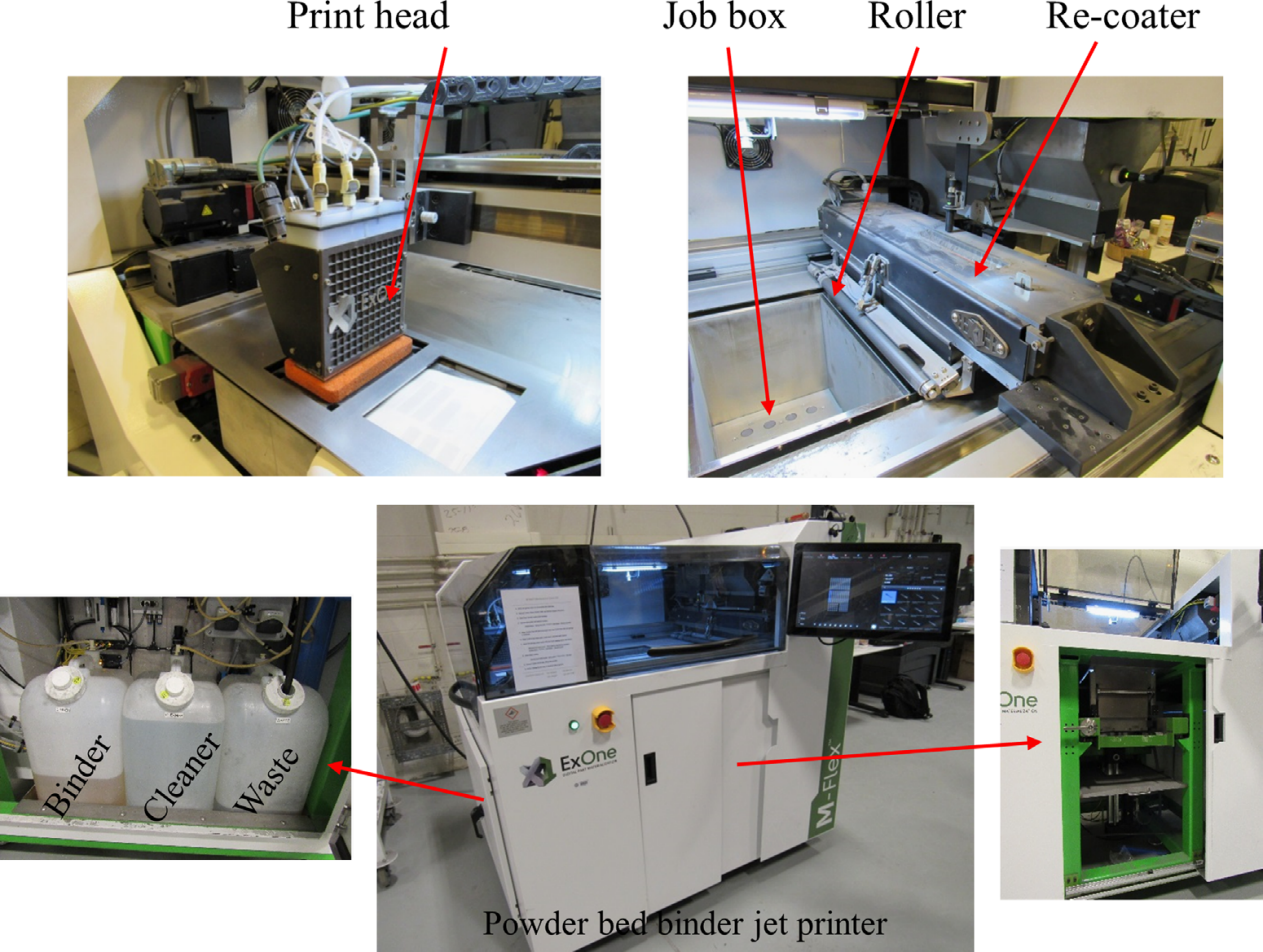
M-Flex ExOne printer used to manufacture samples. Main parts are labeled in the images.

**Fig. 3:**
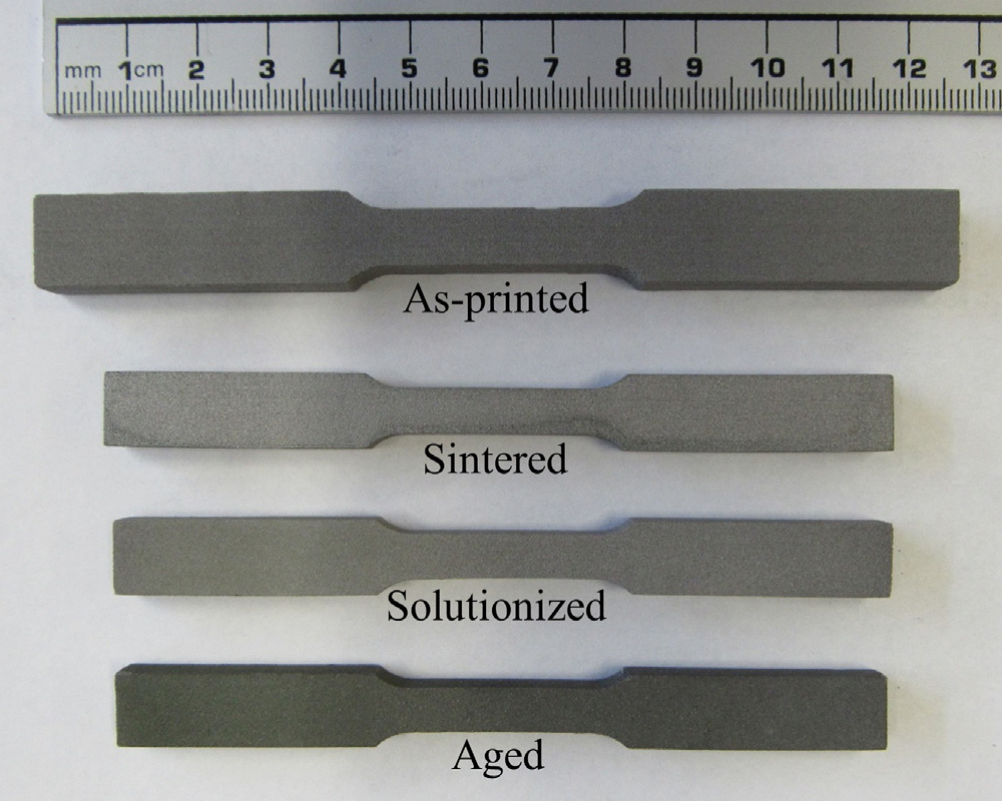
Photograph of a dogbone samples of the BJP alloy 625 parts and then differently heat treated.

**Fig. 4:**
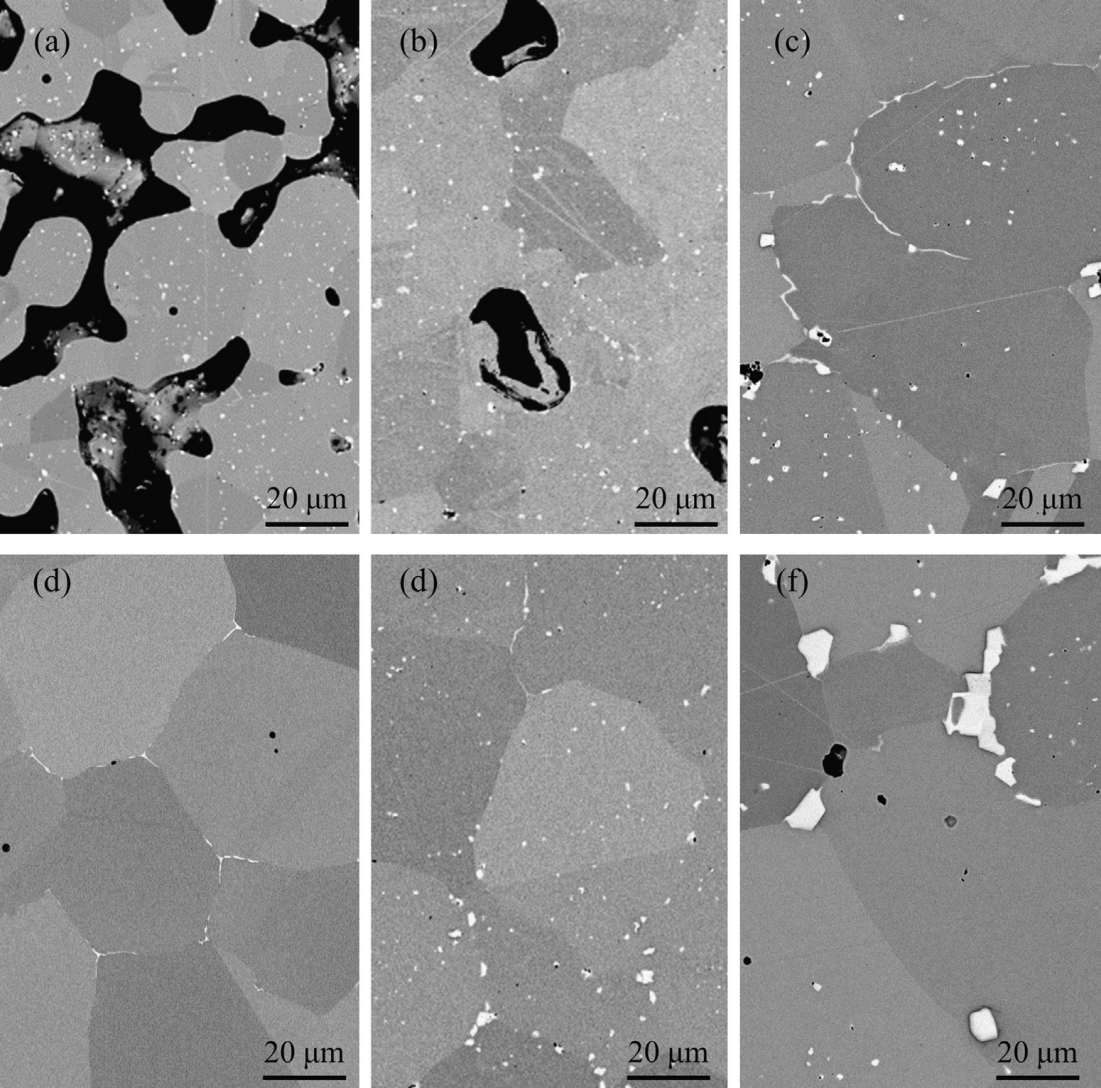
SEM micrographs taken from the cross-section of the BJP alloy 625 samples sintered at (a) 1200 °C, (b) 1240 °C, (c) 1280 °C. The fully densified sample is then heat treated and cross sectional SEM micrographs are (d) solution treated at 1150 °C for 2 h, (e) aged at 745 °C for 20, (f) aged at 745 °C for 60 h.

**Fig. 5:**
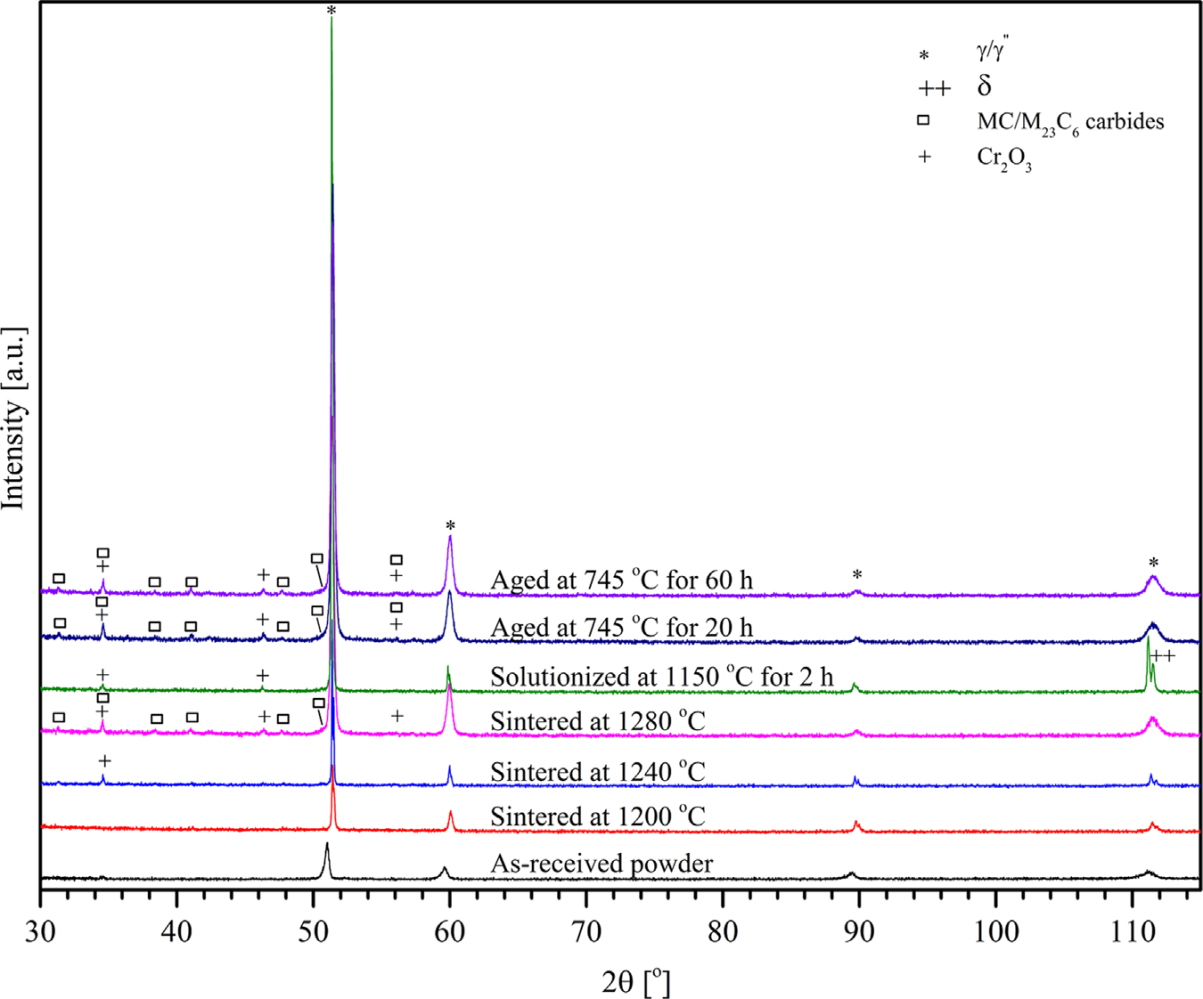
The XRD patterns of the BJP alloy 625 samples sintered at different temperatures and then heat treated at different conditions.

**Fig. 6:**
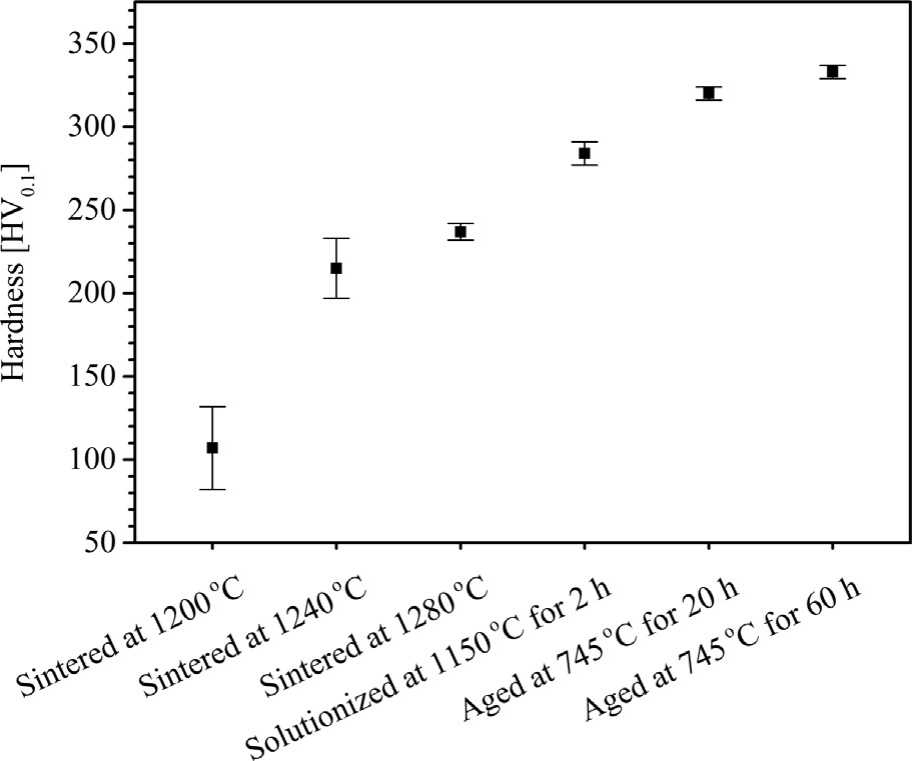
Microhardness values of the BJP alloy 625 samples sintered at different temperatures for 4 h and then differently heat treated.

**Fig. 7:**
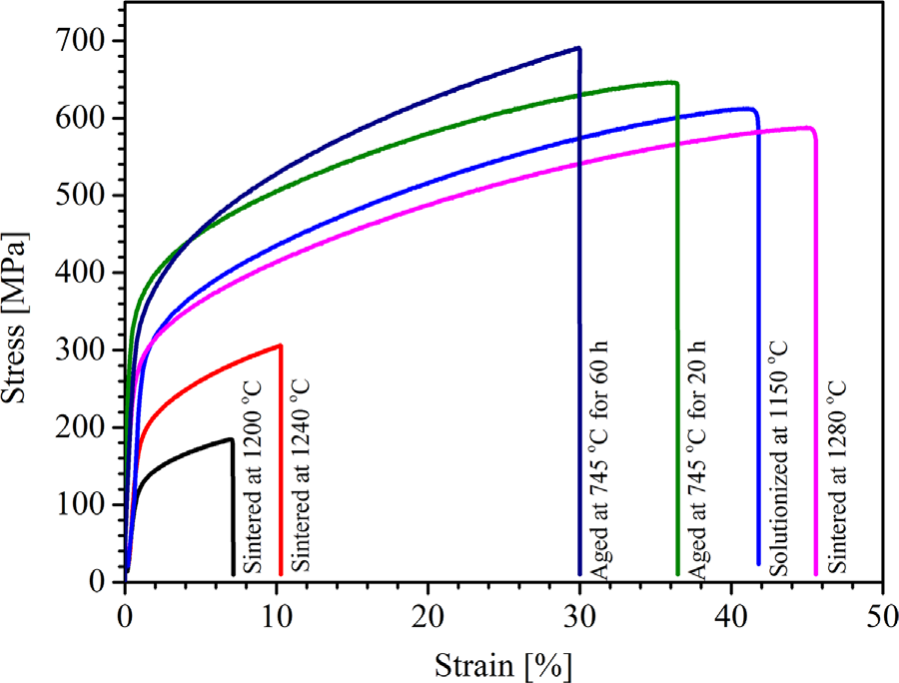
Stress–strain curves of the differently heat treated BJP alloy 625 samples.

The presented data in this paper (see Fig. 1, Fig. 2, Fig. 3, Fig. 4, Fig. 5, Fig. 6, Fig. 7) can be divided into two parts: (1) characterization of the argon atomized powder, and (2) differently heat treated BJP alloy 625 samples in terms of microstructural observations using SEM, phase analysis using XRD, and mechanical testing including microhardness and tensile tests. The data included in this paper is based on experimental results provided in two publications from the authors [1], [2].

## 2 Experimental design, materials and methods

Summary of the obtained data for the argon atomized alloy 625 powder are shown in Fig. 1. The presented data includes powder morphology data obtained using SEM (Fig. 1a–c), particle size distribution (Fig. 1d), DSC-TGA curves (Fig. 1e) and elemental analysis data (Fig. 1f).

The alloy 625 powder (Carpenter Technology Corporation) was spherical in shape as created via vacuum melted argon atomization method. The powder possessed a size distribution between 14 μm and 65 μm with the average particle size of 31 μm.

Fig. 2 illustrates the M-Flex ExOne printer used to manufacture 3-dimensional samples and different parts are labeled as shown in Fig. 2. To additively manufacture dogbone samples, the following printing parameters were used: recoat speed of 130 mm/s, oscillator speed of 2050 rpm, roller speed of 250 rpm, roller traverse speed of 15 mm/s, and drying speed of 17 mm/s, with the thickness for each printing layer of 100 μm. Total number of printed layers is 125. The used binder is made of Ethylene Glycol Monomethyl Ether and Diethylene Glycol. The cleaner is 2-butoxyethanol.

Fig. 3 shows the optical images taken from the as-printed, sintered, solutionized and aged samples. Dimensions of the samples were: (1) as-printed sample (green part): 125 mm long, 12.5 mm wide, 7.5 mm thick, gage length of 29 mm, and (2) differently heat treated samples (sintered, solutionized and aged): 105.7 mm long, 10.4 mm wide, 6 mm thick, gage length of 26 mm. Sintering and different heat treatments were conducted on the printed samples based on the parameters given in our previous studies [1], [2].

The microstructure of the sintered and differently heat treated samples were observed using SEM working with an accelerating voltage of 20 kV as data shown in Fig. 4. Samples were cut using wire saw, mounted, polished and etched with a Kallings solution.

X-ray diffraction pattern was conducted to acquire data for phase formation after sintering and differently treatment conditions. X-ray diffraction (XRD) was performed on a PANanalytical EMPYREAN diffractometer with a Co Kα radiation source (*λ*=1.79 Å, 40 kV, 40 mA) and 2*θ* scans from 30° to 115° and collected data is shown in Fig. 5.

Microhardness measurement was conducted on Leco LM 800 instrument using 100 gf and 10 s dwell time on cross sections of the sintered, solution treated and aged samples and obtained data was presented in Fig. 6. Average of 10 data points were reported. Moreover, tensile test curves were collected using an MTS 880 machine with rate controlled tensile test at 5 mm/min and obtained data are illustrated in Fig. 7.
